# Nursing and Planetary Health: Learning from the Plant Metaphor for Environmental Justice

**DOI:** 10.1590/0034-7167-2025-0288

**Published:** 2026-03-30

**Authors:** Helena Maria Scherlowski Leal David

**Affiliations:** I Universidade do Estado do Rio de Janeiro. Rio de Janeiro, Rio de Janeiro, Brazil

**Keywords:** Nursing, Nursing as a Social Practice, Planetary Health, Environmental Justice, Environmental Health., Enfermería, Enfermería como Práctica Social, Salud Planetaria, Justicia Ambiental, Salud Ambiental.

## Abstract

**Objectives:**

to present a reflection on the relationship between nursing as a social practice and planetary health based on the concepts of civilizing hybris and a plant metaphor.

**Methods:**

theoretical-conceptual discussion, based on criticisms of anthropocentrism and the Anthropocene concept to explain environmental and climate impacts, proposing the idea of a plant metaphor, originating from Stefano Mancuso’s studies, as a provocative element in the debate regarding nursing role and new ways of dealing with the complexity of the topic, breaking with civilizational hybris.

**Results:**

nursing, as a social practice, is considered relevant for proposing expanded actions aimed at environmental justice and planetary health.

**Final Considerations:**

adopting a plant metaphor can reframe our worldview and professional practice as a possible guide for cooperative, non-exploitative care of the planet.

## RESUMEN

**Objetivos:**

reflexionar sobre la relación entre la enfermería como práctica social y la salud planetaria, a partir de los conceptos de hybris civilizatoria y la metáfora de plantas.

**Métodos:**

discusión teórica y conceptual, basada en críticas al antropocentrismo y al concepto del Antropoceno para explicar los impactos ambientales y climáticos, proponiendo la idea de una metáfora de plantas, proveniente de los estudios de Stefano Mancuso, como elemento provocador en el debate sobre el rol de la enfermería y nuevas formas de abordar la complejidad del tema, rompiendo con la hybris civilizatoria.

**Resultados:**

se considera que la enfermería, como práctica social, tiene relevancia para proponer acciones ampliadas orientadas a la justicia ambiental y la salud planetaria.

**Consideraciones Finales:**

adoptar la metáfora de las plantas puede replantear nuestra cosmovisión y práctica profesional como una posible guía para el cuidado cooperativo y no explotador del planeta.

## INTRODUCTION

The term “Anthropocene”, coined by chemist Paul Crutzen and biologist Eugene Stoermer in the early 2000s, proposes a radical break in geological periodization: humanity has become a geophysical force capable of altering Earth systems on a planetary scale, marking the end of the Holocene (the last 11,700 years). Supported by evidence such as atmospheric CO₂ accumulation, ocean acidification, and the accelerated erosion of biodiversity, the concept has gained traction as a metaphor for contemporary ecological crises, although a conceptual debate persists within the field of earth sciences that extends to other disciplinary areas^(1)^.

A central criticism of the Anthropocene lies in its “homogenization of human agency”. Authors such as Jason W. Moore and Donna Haraway^(1)^ argue that the term obscures historical and geopolitical inequalities, treating “humanity” as a monolithic bloc, simplifying, and glossing over complex processes. In reality, we know that environmental impacts are unevenly distributed: while the richest 1% of the population emits more than twice as much CO₂ as the poorest 50%, peripheral, Indigenous, and Global South communities suffer disproportionately from droughts, floods, and land loss^(2)^. For Moore, the emphasis on the “Capitalocene”-an era shaped by the expansionist logic of capitalism-better reveals how exploitation of humans and nonhumans is intertwined, questioning the neutrality of the term “Anthropocene”^(1)^. Furthermore, decolonial studies highlight that Anthropocene narrative often reproduces epistemicide, marginalizing traditional knowledge that has practiced sustainable ways of coexisting with nature for millennia^(3)^.

The Anthropocene concept also faces questions about its intrinsic anthropocentrism. Philosophers like Anna Tsing^(3)^ challenge the idea that humans are the only agents capable of transforming the planet, highlighting the agency of non-human species (such as microorganisms, plants, and fungi) in shaping ecosystems. The very notion of human “domination”, they argue, reinforces a biological hierarchy that ignores the vital interdependence between species. Thus, while the term “Anthropocene” is useful for denouncing the escalating crisis, it remains an “imperfect tool,” requiring transdisciplinary dialogues that integrate climate justice, epistemological pluralism, and a biocentric ethic capable of reimagining our relationship with the Earth beyond the *hubris* of civilization and control.

The term *hybris* (also spelled *húbris* in Portuguese), originating from Greek, was used in tragedy and philosophy to designate the excessive arrogance and ambition of men before the gods, resulting in divine punishments and disorder^(4)^. It has been reinterpreted in debates on civilization, progress, and cultural pluralism to indicate the human belief in superiority over nature and other beings. It is also a critique of the epistemes derived from Enlightenment thought, in which the mind-body dichotomy takes root and weaves ways of thinking about the natural world as an inexhaustible store of wealth to be exploited, which, in turn, serves the capitalist and predatory mode of accumulation^(3)^.

The idea of planetary health is also subject to this criticism, since, in its current form, it is less an ethical revolution and more a symptom of the *hybris* that has brought us to Anthropocene. Its greatest danger lies in making us believe that small technical adjustments-more efficiency, more monitoring, more management-will be enough to avoid collapse without requiring a radical transformation of our civilizational priorities^(1)^.

An important counterpoint in this discussion is the idea of the plant metaphor, developed by Italian botanist Stefano Mancuso in a series of works that explore the results of studies on plant neurobiology and the plant world’s ability to respond and communicate among peers and with other species, and even to move in space, in a notion of long time^(5,6)^. The plant kingdom, which possesses a type of non-centralized cognition and intelligence that challenges civilizational *hubris*, reframes the debate in new terms, breaking with the human-nature dichotomous model of anthropocentrism. Its discoveries not only challenge entrenched biological hierarchies but also question the assumptions that guide our relationship with the planet.

It is important to situate nursing as a social practice and a profession dedicated to defending life at all levels and possibilities. According to key documents from international organizations, nursing is recognized as a strategic pillar for planetary health, defined as integration of human health, ecosystems, and socio-environmental justice. Exploring new paradigms is not only necessary but urgent, given the acceleration of environmental degradation processes worldwide. It is noteworthy that, as a field of care production, modern nursing has advanced based on diverse, historically determined theoretical and epistemological approaches that have sought, in each time and context, to develop the best responses to threats to life and health.

Could the plant metaphor be a way to envision new ways of relating to other living beings, with the central objective of producing and sustaining life in its most integral and powerful forms? Can we consider that this metaphor can inform the equation between social and biological factors for producing health-disease-care processes? Can nursing, as an involved social practice, also constitute a practice capable of perceiving and caring for worlds inhabited by other beings?

Far from answering these questions, the objective of this article, based on a discussion based on some constituent and regulatory elements of plant and fungal life forms brought to light by Stefano Mancuso’s plant neurobiology, is to provoke reflection on other forms of care that nursing can offer and organize toward environmental justice and planetary health, highlighting its potential in various care settings. A broadened and expanded care is directed toward other worlds that constitute our reality and our surroundings, to which we may not be sufficiently attentive.

To this end, we propose reflecting on the limits of civilizing *hybris* to advance towards social and environmental justice, and we recover central aspects related to what Mancuso calls the plant metaphor as an example and possibility of paradigmatic change in what nursing does, pointing out new questions based on this discussion.

### Civilizing *hybris* and control *hybris:* until when?

The roots of civilizational *hybris* are intertwined with the colonial-capitalist project, which transformed socio-ecological relations into commodities. Moore emphasizes that the Capitalocene-the era of capital as the driving force behind environmental change-best explains the current crisis, as accumulation of wealth depended on the simultaneous exploitation of human labor and natural resources. The Green Revolution, for instance, illustrates this dynamic: by prioritizing monocultures and agrochemicals, it increased productivity but degraded soils and excluded traditional knowledge. Technoscience, although presented as a solution, often reinforces the *hybris* of control by promoting false promises, such as climate geoengineering, without questioning unsustainable consumption patterns^(1)^.

In the contemporary ecological context, this *hybris* manifests itself in the belief that humans can (and should) control, dominate, and exploit natural systems without catastrophic consequences, ignoring the fundamental interdependence between human life and ecosystems. The notion that humanity is “outside” or “above” nature, as omnipotent stewards of a planet at our disposal, is an anthropocentric illusion criticized throughout history. This view ignores that we are “symbionts” in multispecies networks^(1)^, in which human survival depends on interdependencies such as pollination^(5)^ and carbon cycle regulation^(2)^. The disruption of these balances exposes the civilizing *hybris* of a project that treats the Earth as a “resource”, not as living tissue of which we are threads^(1)^. As Ailton Krenak^(7)^ summarizes, “We are not the owners of the Earth; we are the Earth trying to understand itself” - an invitation to abandon the fantasy of control and recognize our condition as “dance partners” in systems that transcend domination.

The consequences are catastrophic and unequally distributed. Environmentally, the planet faces global warming, the sixth mass extinction, and widespread pollution, with tipping points, such as the accelerated melting of Greenland, threatening ecosystem stability. Socially, marginalized communities, especially in the Global South, suffer disproportionately from droughts, floods, and loss of territory^(1)^. From the perspective of the *hybris* of control, the illusion of control persists, impeding effective responses by maintaining a false dichotomy between culture and nature. Overcoming *hybris* requires a biocentric shift based on ethics such as *Buen Vivir*, of the Andean *Aymara* peoples, and political ecology, which recognize the vital interdependence between species and the urgency of socio-environmental justice.

The *hubris* of control is not only a philosophical error, but a civilizational pathology that threatens Earth’s habitability. To ensure planetary health, it is urgent to replace the logic of control with an ethics of care, in which humility and cooperation guide our relationship with the living world.

Therefore, the question is: are plants and their modes of existence and resistance capable of teaching us how to respond to these challenges?

### The plant metaphor: lessons from plant neurobiology

Stefano Mancuso develops the plant metaphor as “intelligent and social organisms”^(6)^. He compares the structure of plants to a “decentralized and cooperative society”, highlighting that their “intelligence” is not centralized (like a brain), but distributed in networks - an implicit critique of anthropocentrism and human hierarchy:

*Plants are a nation.* [...] *A nation without command centers, without pyramids of power, without armies, without borders, in which each individual is free to follow their own path, but always for the benefit of the community.* [...] *While we humans pride ourselves on our intelligence, plants have already invented the future^(6)^* (free translation).

Mancuso uses the image of a “nation” to contrast plant organization (cooperative, sustainable, and resilient) with human civilizational *hubris*-based on domination, competition, and resource exploitation. By describing plants as beings capable of solving complex problems without hierarchies (e.g., root networks that function as a “plant internet”), he challenges the notion of human superiority and proposes an alternative model of coexistence.

The notion of distributed intelligence in plants, which operates without a central organ and emerges from communication between cells in the roots, resembles an integrated nervous system. This network allows plants, for instance, to engage in chemical and electrical communication: roots emit signals to warn of imminent threats, such as herbivore attacks, or to indicate the location of essential resources, such as water and nutrients. Furthermore, plants demonstrate collective decision-making, assessing environmental variables such as light intensity, soil moisture, and competition for space to strategically direct their growth, avoiding toxic areas and prioritizing nutrient-rich regions. This process is amplified by the so-called “plant internet”, an underground network formed by symbioses between roots and fungi (mycorrhizae) that allows for the sharing of information and resources at scale. Popularized by ecologist Suzanne Simard as the “Wood Wide Web”, this web connects individuals of the same species and even of different species, transforming entire ecosystems into collaborative and interdependent systems. Thus, plant intelligence not only challenges the notion of biological centrality, but reveals an adaptive sophistication that redefines our understanding of life and cooperation in nature^(5,6)^.

If plants possess “agency”-the ability to act and respond to the environment with adaptive strategies-practices like deforestation and monocultures become morally questionable, as they assume that nature is a passive object to be dominated. This logic ignores that plants are sentient beings, capable of complex communication, collective decision-making, and even altruism, like trees that nurture young seedlings through underground networks^(6)^.

By adopting a biocentric ethic, which recognizes the intrinsic value of all life forms (not just their utilitarian use), practices such as cutting down ancient trees-which store centuries of ecological knowledge-or contaminating soil with pesticides are revealed as forms of “ecological violence”. This violence not only disrupts ecosystems but also threatens human survival, since we depend on environmental services such as pollination and climate regulation.

As an alternative, Mancuso proposes models inspired by plant cooperation, such as agroforestry and regenerative agriculture. These systems mimic the distributed intelligence of plants, maintaining natural nutrient cycles, biodiversity, and resilience^(6)^. While monocultures deplete the soil in just a few years, agroforests-where complementary species grow together-reproduce the mutualistic logic of forests, demonstrating that it is possible to produce food without denying the agency of plant life.

Mancuso, paradoxically, has been using science to show that the human claim to resolve and control climate and environmental challenges is naive: plants, even without brains, have already solved problems that our technology cannot (e.g., energy efficiency, symbiosis). What the plant metaphor demands of us is the humility and generosity to recognize that we cooperate less than plants, since, while humans compete for resources, plant networks like mycorrhizae operate in symbiosis. We are, therefore, dependent on “inferior” beings, since, without photosynthesis, there would be no oxygen or food.

Thus, recognizing plants’ sentience is not only a scientific revolution, but an ethical imperative to rethink our relationship with the planet.

### Nursing as a caretaker of worlds: expanding practices, confronting inequalities

Recognizing nursing as a social practice, more than a profession or occupation, presupposes that caring for people requires mapping and addressing structural inequalities. In Anthropocene, this struggle is amplified, as caring for humans is inseparable from caring for ecosystems that sustain them. Examples include nurses’ work caring for riverside populations in the Amazon who, in combating malaria outbreaks, must be aware that distributing mosquito nets is not enough; it is necessary to challenge the deforestation that increases mosquito proliferation and destroys traditional ways of life. Or, in the peripheries affected by floods, nursing care requires coordinating sanitation and housing policies, recognizing that vulnerability to “natural” disasters is socially constructed. Nurses working in these contexts are necessarily grassroots educators, whose work is neither voluntaristic nor spontaneous. This is an intentional and pedagogically qualified professional action around Popular Education in Health proposals, which is embodied in the current Brazilian National Policy of Popular Education in Health in the Unified Health System (In Portuguese, *Política Nacional de Educação Popular em Saúde no Sistema Único de Saúde* - PNEPS-SUS).

Internationally, it is important to explicitly recognize, over the last decade, the role of nurses not only in addressing threats directly related to human illness, but also as important social actors capable of integrating diverse initiatives, anchored in a pluralistic and interdisciplinary perspective, in favor of environmental health and in leading educational processes. The United Nations, in 2020, highlighted that nurses represent the majority of healthcare professionals in the world, working on the front lines of health and environmental crises, and in promoting community health, advocating for investments in nursing to strengthen health systems resilient to climate change and inequalities^(8)^. International and national actions, based on the “Nursing Now” campaign, proposed by the World Health Organization, between 2018 and 2023, brought visibility to nursing and converged, during the pandemic, to highlight the importance of nursing in the face of serious threats to global health^(9)^. In 2023, the International Council of Nurses positioned nurses as agents of socio-environmental transformation capable of mitigating ecological damage (e.g., reducing hospital waste) and promoting “green health”. It also emphasized their role in community education on environmental determinants of health, such as pollution, water scarcity, and others^(10)^. This same agency, when updating the global definition of nurse and nursing in 2025, brought about an important discursive shift by presenting nursing as, in addition to a care profession, a social force defending universal human rights, including terms such as environmental justice, equity, sustainability, and ethical commitment.

Recognizing and understanding the complex processes that result in planetary health, in which nursing can and should be present, goes beyond an abstract or merely academic idea. It involves, within professional practice, strengthening participatory, dialogically constructed actions, anchored in paradigms that favor the development of new care and educational perspectives. In Brazilian Primary Health Care (PHC) territories, for instance, nurses’ educational actions need to break with traditional hygienist practices to advance a collective understanding of socio-environmental determinants, as currently proposed by PNEPS-SUS and other health policies developed in territories marked by cultural and historical diversity, such as *quilombos* (translator’s note: community formed in Brazil by people who fled slavery and built a life of freedom), indigenous lands, riverside and coastal regions, among others.

Here, we highlight the misconception that adopting so-called “active” teaching and learning methodologies alone can bring about sustainable paradigm shifts. Professionals must constantly ask themselves their role in the face of threats to water quality, fires, deforestation, and other ongoing environmental devastation, through the following questions: will we simply observe the damage, or can we propose dialogic educational processes with the affected populations, understanding the threats as problem situations for which there is no single answer? How can we respond to this ethical-political call? How can we, as a collective, guide our representative bodies and administrators in formulating and defending public policies focused on the environment and health?

Based on these questions, in the examples cited and in many other everyday situations, nursing is able to practice the ecology of care, challenging the *hybris* that separates humans from nature. Its social role includes denouncing that indigenous health, for instance, depends on the preservation of territories threatened by agribusiness-a connection that the traditional biomedical model ignores.

Nursing, historically linked to individual care, is called upon to expand its role in the 21^st^ century to a practice that cares for worlds, integrating human health, ecology, and social justice, and breaking with anthropocentric *hybris* in favor of an ecocentric perspective. This approach recognizes that individuals’ health is intrinsically linked to ecosystems’ health, requiring actions that transcend the traditional biomedical model.

Returning to Mancuso’s plant neurobiology and the forms of plant intelligence and collective action, if we accept this non-human agency, how can nursing integrate this knowledge? The answer lies in redefining care not only as a “monologue between species” (humans caring for humans), but as a dialogue of care with the environment and among diverse social groups. We return here to the essentially educational role of nursing. Nursing educates when it cares for patients, prevents illness, and promotes health by defending vulnerable populations at all levels of care. Despite this, nursing work has a greater immediate potential in PHC.

We need to understand and incorporate local experiences of ecocentric environmental defense practices as part of nursing’s work: where are the professionals who, for instance, teach communities to preserve water sources, recognizing that water health is vital to preventing disease? Where are the professionals who support movements and fight for laws that recognize rivers or forests as subjects of rights, understanding that their “health” is a prerequisite for our own?

Despite this potential, nursing faces structural obstacles to achieving this vision, such as: work overload, with professionals overwhelmed by care demands and little room for environmental policy action; and technical training, through curricula that still prioritize clinical procedures over topics such as climate justice or political ecology. Commodification of care, which reduces health to an individual service and erases its ecological dimension, is at the root of the issues preventing nursing from expanding its practice in an ecocentric direction.

Anthropocene’s *hybris* has taught us that there is no human health on a sick planet. It is up to nursing, as a social practice, to contribute to “sewing” this rupture, remembering that every act of care, with every vaccination campaign, every gesture of welcome, is also an act of resistance against a system that poisons rivers, soils, and bodies. If *hybris* is the disease, nursing can be, in communion and collective dialogue with other professionals and other beings, a caretaker of worlds.

While “healing” may imply a power relationship (human over nature), “caring” presupposes humility and shared responsibility. Nursing, as a caretaker of worlds, does not seek to “save” the planet, but to weave networks of coexistence that sustain life in all its forms.

This approach echoes Donna Haraway’s thinking, who advocates “staying with the trouble”, i.e., recognizing the issues and engaging in daily practices of care, even without guaranteed solutions, in the face of serious environmental threats. Staying with the problem means establishing connections with other people, disciplines, and other beings, establishing “new kinships”. The author also emphasizes the importance of sustaining the creativity of people who care and take action, including activists, artists, scientists, and communities^(11)^.

According to indigenous leader Ailton Krenak’s words, “we do not own the Earth; we are its extension”^(7)^. Nursing, by embracing this maxim, can redefine its legacy: from a profession that cares for humans to a movement that defends life in all its forms.

## FINAL CONSIDERATIONS

This text does not seek answers, but rather provokes reflection based on a few questions: how can we balance the urgency of planetary health with the need to deconstruct anthropocentrism? If plants think, who are we to decide their fate? Mancuso’s science is a mirror that reflects our *hybris*, and perhaps, in this reflection, we find the humility necessary to re-exist.

Adopting a plant metaphor can reframe our worldview and professional practice as a possible guide for cooperative, non-exploitative care and for environmental justice. As nurses, as we seek to embrace this perspective in our daily practice, we must have the humility to engage in necessary and continuous learning, expanding our listening skills. This implies taking over our permanent role as grassroots educators with a permanent, dialogical perspective, seeking greater qualifications on planetary health issues and sharing our local initiatives, especially those developed through dialogue in the different territories where we operate. Building on our existing leadership as mediators between healthcare professionals, organizations, educators, and trainers, we must address the various forms of social injustice, including environmental injustice.

We agree with the authors featured in this reflection, who assert that there’s no point in declaring war on global warming if we continue to wage war against the Earth itself. True healing will not come from management plans, but from an ethical awakening that reminds us: we are guests, not owners, of this planet. And perhaps the most urgent lesson is to learn to listen to, rather than command, the Earth’s silenced voices-including those that have never been interested in saving us, but certainly have and will play a central role in the future of our species-plants.

## Data Availability

Not applicable.
