# Supplementary material for: Nursing and Planetary Health: Learning from the Plant Metaphor for Environmental Justice
Source: Rev Bras Enferm. 2026 Mar 30;79:e20250288. doi: 10.1590/0034-7167-2025-0288 (PMC13053010; doi:10.1590/0034-7167-2025-0288)
Supplement: Translation

# Translation: Enfermagem e Saúde Planetária: Aprendendo com a Metáfora das Plantas para a Justiça Ambiental

**DOI:** 10.1590/0034-7167-2025-0288pt

**Authors:** Helena Maria Scherlowski Leal David

**Affiliations:** I Universidade do Estado do Rio de Janeiro. Rio de Janeiro, Rio de Janeiro, Brasil

**Keywords:** Enfermagem, Enfermagem como Prática Social, Saúde Planetária, Justiça Ambiental, Saúde Ambiental.

## Abstract

**Objetivos:**

apresentar uma reflexão sobre a relação entre a enfermagem como prática social e a saúde planetária com base nos conceitos de *hybris* civilizatória e na metáfora das plantas.

**Métodos:**

discussão teórico-conceitual, a partir de críticas ao antropocentrismo e ao conceito de Antropoceno para explicar os impactos ambientais e climáticos, propondo a ideia de metáfora das plantas, oriunda dos estudos de Stefano Mancuso, como elemento provocador do debate a respeito do papel da enfermagem e sobre novas formas de lidar com a complexidade do tema, rompendo com a *hybris* civilizatória.

**Resultados:**

considera-se que a enfermagem, como prática social, possui relevância para propor ações ampliadas com vistas à justiça ambiental e à saúde planetária.

**Considerações Finais:**

adotar a metáfora das plantas pode reconduzir nossa visão de mundo e de prática profissional como um possível guia para um cuidado cooperativo e não exploratório do planeta.

## INTRODUÇÃO

O termo “Antropoceno”, cunhado pelo químico Paul Crutzen e pelo biólogo Eugene Stoermer, no início dos anos 2000, propõe uma ruptura radical na periodização geológica: a humanidade teria se tornado uma força geofísica capaz de alterar os sistemas terrestres em escala planetária, marcando o fim do Holoceno (últimos 11,7 mil anos). Apoiado em evidências como o acúmulo de CO₂ atmosférico, a acidificação dos oceanos e a erosão acelerada da biodiversidade, o conceito ganhou força como metáfora das crises ecológicas contemporâneas, embora persista um debate conceitual interno ao campo das ciências da terra que se estende a outras áreas disciplinares^(1)^.

Uma crítica central à ideia do Antropoceno reside em sua “homogeneização da agência” humana. Autores como Jason W. Moore e Donna Haraway^(1)^ argumentam que o termo obscurece as desigualdades históricas e geopolíticas, tratando a “humanidade” como um bloco monolítico, simplificando e escamoteando processos complexos. Na realidade, sabemos que os impactos ambientais estão desigualmente distribuídos: enquanto a parcela de 1% mais rica da população emite mais do que o dobro de CO₂ que a parcela de 50% mais pobre, comunidades periféricas, indígenas e do Sul Global sofrem desproporcionalmente com secas, enchentes e perda de territórios^(2)^. Para Moore, a ênfase no “Capitaloceno” - uma era moldada pela lógica expansionista do capitalismo - revela melhor como a exploração de humanos e não humanos está entrelaçada, questionando a neutralidade do termo “Antropoceno”^(1)^. Além disso, estudos decoloniais destacam que a narrativa do Antropoceno muitas vezes reproduz um epistemicídio, marginalizando saberes tradicionais que há milênios praticam modos sustentáveis de coexistência com a natureza^(3)^.

O conceito de Antropoceno enfrenta também questionamentos sobre seu antropocentrismo intrínseco. Filósofos como Anna Tsing^(3)^ desafiam a ideia de que os humanos são os únicos agentes com capacidade de transformar o planeta, destacando a agência de espécies não humanas (como microrganismos, plantas e fungos) na conformação de ecossistemas. A própria noção de “dominação” humana, segundo eles, reforça uma hierarquia biológica que ignora a interdependência vital entre espécies. Assim, o Antropoceno, embora útil para denunciar a escalada da crise, permanece sendo uma “ferramenta imperfeita”, exigindo diálogos transdisciplinares que integrem justiça climática, pluralismo epistemológico e uma ética biocêntrica capaz de reimaginar nossa relação com a Terra, além da *hybris* civilizatória e, no seu rastro, da *hybris* do controle.

O termo *hybris* (também usado com a grafia húbris, em língua portuguesa), oriundo do grego, era usado na tragédia e filosofia para designar a arrogância e a ambição desmedidas dos homens diante dos deuses, tendo como consequências os castigos divinos e desordem^(4)^. Vem sendo ressignificado, nos debates sobre civilização, progresso e pluralismo cultural, para indicar a crença humana de superioridade sobre a natureza e os outros seres. Trata-se de uma crítica também às epistemes tributárias do pensamento iluminista, nas quais a dicotomia mente-corpo se enraíza e tece formas de pensar o mundo natural como estoque inesgotável de riqueza a ser explorada, o que, por sua vez, está a serviço do modo capitalista e predatório de acumulação^(3)^.

A ideia de saúde planetária também está sujeita a esta critica, já que, em sua forma atual, é menos uma revolução ética e mais um sintoma da *hybris*, que nos trouxe ao Antropoceno. Seu maior perigo está em nos fazer acreditar que pequenos ajustes técnicos - mais eficiência, mais monitoramento, mais gestão - serão suficientes para evitar o colapso sem exigir a transformação radical de nossas prioridades civilizatórias^(1)^.

Um contraponto importante nesta discussão é a ideia da metáfora das plantas, desenvolvida pelo botânico italiano Stefano Mancuso na sequência de obras que exploram resultados de estudos a respeito da neurobiologia das plantas e da capacidade do mundo vegetal de responder e comunicar entre pares e com outras espécies, e até mesmo de se mover no espaço, em uma noção de tempo longo^(5,6)^. O reino vegetal, que possui um tipo de cognição e de inteligência não centralizada que desafia a *hybris* civilizatória, recoloca o debate em outros termos, rompendo com o modelo dicotômico homem-natureza do antropocentrismo. Suas descobertas não apenas desafiam hierarquias biológicas arraigadas, mas também questionam os pressupostos que orientam nossa relação com o planeta.

Aqui, é importante situar a enfermagem como prática social e profissão voltada à defesa da vida em todos os seus níveis e possibilidades. Segundo documentos-chave de organizações internacionais, a enfermagem é reconhecida como pilar estratégico para a saúde planetária, explicitada como a integração entre saúde humana, ecossistemas e justiça socioambiental. Explorar novos paradigmas é não apenas necessário, mas urgente, diante da aceleração dos processos de degradação ambiental em todo o mundo. Ressalta-se que, como campo de produção do cuidado, a enfermagem moderna avançou a partir de abordagens teóricas e epistemológicas plurais, historicamente determinadas e que buscaram, em cada tempo e contexto, construir as melhores respostas às ameaças à vida e à saúde.

Seria a metáfora das plantas uma forma de pensar novas maneiras de nos relacionarmos com outros seres vivos, tendo como objetivo central a produção e manutenção da vida nas suas formas mais integrais e potentes? Podemos considerar que essa metáfora pode compor a equação entre fatores sociais e biológicos para produção dos processos de saúde-doença-cuidado? Pode a enfermagem, como prática social implicada, constituir-se também em uma prática capaz de enxergar e cuidar de mundos habitados por outros seres?

Longe de responder a tais questões, o objetivo deste artigo é, a partir de uma discussão calcada em alguns elementos constituintes e reguladores das formas de vida vegetais e fúngicas trazidos pela neurobiologia das plantas de Stefano Mancuso, provocar uma reflexão acerca de outras formas de cuidado que a enfermagem é capaz de ofertar e organizar na direção da justiça ambiental e da saúde planetária, ressaltando sua potencialidade nos vários cenários de cuidado. Um cuidado ampliado e alargado é dirigido a outros mundos que constituem nossa realidade e nosso entorno em relação aos quais talvez não estejamos suficientemente atentos.

Para isso, propomos refletir sobre os limites da *hybris* civilizatória para avançar rumo à justiça social e ambiental, e recuperamos aspectos centrais relativos ao que Mancuso denomina metáfora das plantas como exemplo e possibilidade de mudança paradigmática no que-fazer da enfermagem, apontando novas questões a partir desta discussão.

### A *hybris* civilizatória e a *hybris* do controle: até quando?

As raízes da *hybris* civilizatória estão entrelaçadas ao projeto colonial-capitalista, que transformou relações socioecológicas em mercadorias. Moore destaca que o Capitaloceno - a era do capital como força motriz das mudanças ambientais - explica melhor a crise atual, pois a acumulação de riqueza dependeu da exploração simultânea de trabalho humano e recursos naturais. A Revolução Verde, por exemplo, ilustra essa dinâmica: ao priorizar monoculturas e agrotóxicos, aumentou a produtividade, mas degradou solos e excluiu saberes tradicionais. A tecnociência, embora apresentada como solução, muitas vezes reforça a *hybris* do controle, ao promover falsas promessas, como a geoengenharia climática, sem questionar padrões insustentáveis de consumo^(1)^.

No contexto ecológico contemporâneo, essa *hybris* se manifesta na crença de que os seres humanos podem (e devem) controlar, dominar e explorar os sistemas naturais sem consequências catastróficas, ignorando a interdependência fundamental entre a vida humana e os ecossistemas. A noção de que a humanidade está “fora” ou “acima” da natureza, como gestores onipotentes de um planeta à nossa disposição, é uma ilusão antropocêntrica criticada ao longo da história. Essa visão ignora que somos “simbiontes” em redes multiespécies^(1)^, nas quais a sobrevivência humana depende de interdependências como a polinização^(5)^ e a regulação do ciclo do carbono^(2)^. A ruptura desses equilíbrios expõe a *hybris* civilizatória de um projeto que trata a Terra como “recurso”, não como tecido vivo do qual somos fios^(1)^. Como sintetiza Ailton Krenak^(7)^, “Não somos donos da Terra; somos a Terra tentando se entender” - um convite a abandonar a fantasia de controle e reconhecer nossa condição de “parceiros de dança” em sistemas que transcendem a dominação.

As consequências são catastróficas e desigualmente distribuídas. Ambientalmente, o planeta enfrenta o aquecimento global, a sexta extinção em massa e a poluição generalizada, com pontos de não retorno, como o degelo acelerado da Groenlândia, ameaçando a estabilidade ecossistêmica. Socialmente, comunidades marginalizadas, especialmente no Sul Global, sofrem desproporcionalmente com secas, inundações e perda de territórios^(1)^. Na perspectiva da *hybris* do controle, há persistência na ilusão de controle que impede respostas efetivas, pois mantém a falsa dicotomia entre cultura e natureza. Superar a *hybris* exige uma virada biocêntrica baseada em éticas como o *Buen Vivir*, dos povos andinos Aymara, e a ecologia política, que reconheçam a interdependência vital entre espécies e a urgência de justiça socioambiental.

A *hybris* do controle não é apenas um erro filosófico, mas uma patologia civilizatória que ameaça a habitabilidade da Terra. Para garantir a saúde planetária, é urgente substituir a lógica do controle por uma ética do cuidado, em que humildade e cooperação guiem nossa relação com o mundo vivo.

Portanto, questiona-se: seriam as plantas e seus modos de existência e resistência capazes de nos ensinar a responder a esses desafios?

### A metáfora das plantas: lições da neurobiologia vegetal

Stefano Mancuso desenvolve a metáfora das plantas como “organismos inteligentes e sociais”^(6)^. Ele compara a estrutura das plantas a uma “sociedade descentralizada e cooperativa”, destacando que sua “inteligência” não está centralizada (como um cérebro), mas distribuída em redes - uma crítica implícita ao antropocentrismo e à hierarquia humana:

*As plantas são uma nação.* [...] *Uma nação sem centros de comando, sem pirâmides de poder, sem exércitos, sem fronteiras, na qual cada indivíduo é livre de seguir seu próprio caminho, mas sempre em benefício da comunidade.* [...] *Enquanto nós, humanos, nos orgulhamos de nossa inteligência, as plantas já inventaram o futuro^(6)^.*

Mancuso usa a imagem da “nação” para contrastar a organização vegetal (cooperativa, sustentável e resiliente) com a *hybris* civilizatória humana- baseada em dominação, competição e exploração dos recursos. Ao descrever as plantas como seres capazes de resolver problemas complexos sem hierarquias (ex., redes de raízes que funcionam como “internet vegetal”), ele desafia a noção de superioridade humana e propõe um modelo alternativo de coexistência.

A noção de inteligência distribuída nas plantas, que opera sem um órgão central e emerge da comunicação entre células nas raízes, assemelha-se a um sistema nervoso integrado. Essa rede permite, por exemplo, que as plantas realizem comunicação química e elétrica: as raízes emitem sinais para alertar sobre ameaças iminentes, como o ataque de herbívoros, ou para indicar a localização de recursos essenciais, como água e nutrientes. Além disso, as plantas demonstram tomada de decisão coletiva, avaliando variáveis ambientais, como intensidade de luz, umidade do solo e competição por espaço, para direcionar seu crescimento de forma estratégica, evitando áreas tóxicas e priorizando regiões ricas em nutrientes. Esse processo é amplificado pela chamada “internet das plantas”, uma rede subterrânea formada por simbioses entre raízes e fungos (micorrizas) que permite o compartilhamento de informações e recursos em escala. Popularizada pela ecologista Suzanne Simard como “*Wood Wide Web*”, essa rede conecta indivíduos de uma mesma espécie e até de espécies diferentes, transformando ecossistemas inteiros em sistemas colaborativos e interdependentes. Assim, a inteligência vegetal não apenas desafia a noção de centralidade biológica, mas revela uma sofisticação adaptativa que redefine nossa compreensão sobre vida e cooperação na natureza^(5,6)^.

Se as plantas possuem “agência” - capacidade de agir e responder ao ambiente com estratégias adaptativas - práticas como desmatamento e monoculturas se tornam moralmente questionáveis, pois pressupõem que a natureza é um objeto passivo a ser dominado. Essa lógica ignora que plantas são seres sencientes, capazes de comunicação complexa, tomada de decisão coletiva e até altruísmo, como árvores que nutrem mudas jovens através de redes subterrâneas^(6)^.

Ao adotar uma ética biocêntrica, que reconhece o valor intrínseco de todas as formas de vida (não apenas seu uso utilitário), práticas como derrubar árvores centenárias - que armazenam séculos de conhecimento ecológico - ou contaminar solos com agrotóxicos se revelam formas de “violência ecológica”. Essa violência não só desequilibra ecossistemas, mas também ameaça a sobrevivência humana, já que dependemos de serviços ambientais, como polinização e regulação climática.

Como alternativa, Mancuso propõe modelos inspirados na cooperação vegetal, como agroflorestas e agricultura regenerativa. Esses sistemas imitam a inteligência distribuída das plantas, mantendo ciclos naturais de nutrientes, biodiversidade e resiliência^(6)^. Enquanto monoculturas esgotam o solo em poucos anos, as agroflorestas - onde espécies complementares crescem juntas - reproduzem a lógica mutualista das florestas, demonstrando que é possível produzir alimentos sem negar a agência da vida vegetal.

Mancuso, paradoxalmente, vem usando a ciência para mostrar que a pretensão humana de resolução e controle dos desafios climáticos e ambientais é ingênua: as plantas, mesmo sem cérebros, já resolveram problemas que nossa tecnologia não consegue (ex., eficiência energética, simbiose). O que a metáfora das plantas está a exigir de nós é a humildade e generosidade de reconhecer que cooperamos menos do que as plantas, já que, enquanto humanos competem por recursos, redes vegetais como as micorrizas operam em simbiose. Somos, portanto, dependentes de seres “inferiores”, pois, sem a fotossíntese, não haveria oxigênio ou alimentos.

Assim, reconhecer a senciência das plantas não é apenas uma revolução científica, mas um imperativo ético para repensar nossa relação com o planeta.

### Enfermagem cuidadora de mundos: ampliando práticas, enfrentando desigualdades

Reconhecer a enfermagem como prática social, mais do que profissão ou ocupação, pressupõe que cuidar de pessoas exige mapear e enfrentar desigualdades estruturais. No Antropoceno, essa luta se amplia, pois cuidar de humanos é inseparável de cuidar dos ecossistemas que os sustentam. Podemos mencionar como exemplos a prática dos enfermeiros que cuidam de populações ribeirinhas na Amazônia que, no combate aos surtos de malária, devem estar cientes de que não basta distribuir mosquiteiros; é preciso questionar o desmatamento que aumenta a proliferação de mosquitos e destrói modos de vida tradicionais. Ou, ainda, nas periferias afetadas por enchentes, o cuidado de enfermagem exige articular políticas de saneamento e habitação, reconhecendo que a vulnerabilidade a desastres “naturais” é socialmente construída. Enfermeiros que atuam nesses contextos são, necessariamente, educadores populares, cuja atuação não é voluntarista ou espontânea. Trata-se de uma atuação profissional intencional e pedagogicamente qualificada em torno das propostas da Educação Popular em Saúde, que se concretiza na atual Política Nacional de Educação Popular em Saúde no Sistema Único de Saúde (PNEPS-SUS) em curso.

Internacionalmente, é importante explicitar o reconhecimento, na última década, do papel do enfermeiro não apenas diante das ameaças diretamente relacionadas ao adoecimento humano, mas também como importante ator social capaz de integrar iniciativas diversas, ancoradas em uma perspectiva plural e interdisciplinar, em favor da saúde ambiental e na liderança de processos educativos. A Organização das Nações Unidas, em 2020, destacou que enfermeiros representam a maioria dos profissionais de saúde no mundo, atuando na linha de frente de crises sanitárias e ambientais, e na promoção da saúde comunitária, defendendo investimentos na enfermagem para fortalecer sistemas de saúde resilientes às mudanças climáticas e às desigualdades^(8)^. Ações internacionais e nacionais, a partir da campanha “*Nursing Now*”, proposta pela Organização Mundial da Saúde, entre 2018 e 2023, trouxeram visibilidade para a enfermagem e convergiram, durante a pandemia, para ressaltar a importância da enfermagem diante de graves ameaças graves à saúde global^(9)^. Em 2023, o Conselho Internacional de Enfermeiros posicionou enfermeiros como agentes de transformação socioambiental capazes de mitigar danos ecológicos (ex., redução de resíduos hospitalares) e promover “saúde verde”. Enfatizou, ainda, o papel na educação comunitária sobre determinantes ambientais da saúde, como poluição, escassez hídrica, entre outros^(10)^. Este mesmo órgão, ao atualizar a definição global de enfermeiro e enfermagem em 2025, trouxe uma mudança discursiva importante ao apresentar a enfermagem como, além de profissão de cuidado, uma força social de defesa de direitos humanos universais, incluindo termos como justiça ambiental, equidade, sustentabilidade e compromisso ético.

Reconhecer e compreender os complexos processos que resultam em uma saúde planetária e nos quais a enfermagem pode e deve atual vai além de uma ideia abstrata ou apenas acadêmica. Trata-se de, no escopo da prática profissional, fortalecer ações participativas, dialogicamente construídas, ancoradas em paradigmas que favorecem a construção de novas perspectivas assistenciais e educativas. Nos territórios brasileiros da Atenção Primária à Saúde (APS), por exemplo, as ações educativas dos enfermeiros precisam romper com as práticas higienistas tradicionais para avançar em uma compreensão coletiva acerca dos determinantes socioambientais, tal como é hoje proposto pela PNEPS-SUS e outras políticas de saúde desenvolvidas nos territórios marcados pela diversidade cultural e histórica, como quilombos, terras indígenas, regiões ribeirinhas, costeiras, entre outras.

Aqui, chama-se a atenção para o engodo que é entender que a simples adoção das chamadas “metodologias ativas” de ensino-aprendizagem é capaz de, por si só, proporcionar mudanças paradigmáticas sustentáveis. É preciso que os profissionais busquem se perguntar, a todo momento, sobre seu papel diante das ameaças à qualidade das águas, das queimadas, dos desmatamentos e outras ações de devastação ambiental em curso, através das seguintes questões: ficaremos apenas constatando os danos ou podemos propor processos educativos dialógicos com as populações afetadas, entendendo as ameaças como situações-problema para as quais não há uma única resposta? Como responder a esta convocação ético-política? Como podemos, como coletivo, pautar nossos órgãos de representação e gestores para formulação e defesa das políticas públicas que se voltam ao ambiente e à saúde?

A partir dessas questões, nos exemplos citados e em muitas outras situações cotidianas, a enfermagem é capaz de praticar a ecologia do cuidado, desafiando a *hybris*, que separa humanos da natureza. Seu papel social inclui denunciar que a saúde indígena, por exemplo, depende da preservação de territórios ameaçados pelo agronegócio - uma ligação que o modelo biomédico tradicional ignora.

A enfermagem, historicamente vinculada ao cuidado individual, é convocada a expandir seu papel no século XXI para uma prática cuidadora de mundos, integrando saúde humana, ecologia e justiça social, e rompendo com a *hybris* antropocêntrica, em favor de uma perspectiva ecocêntrica. Essa abordagem reconhece que a saúde dos indivíduos está intrinsecamente ligada à saúde dos ecossistemas, exigindo ações que transcendam o modelo biomédico tradicional.

Voltando à neurobiologia das plantas em Mancuso e às formas de inteligência e ação coletiva vegetal, se aceitamos essa agência não humana, como a enfermagem pode integrar esse saber? A resposta está em redefinir o cuidado não apenas como um “monólogo entre espécies” (humano cuidando de humano), mas como um diálogo de cuidado com o ambiente e entre os diversos grupos sociais. Retornamos aqui ao papel essencialmente educativo da ação da enfermagem, que educa quando cuida, quando previne, que promove a saúde quando defende populações vulneráveis, em todos os níveis de atenção, apesar do maior potencial imediato da atuação da enfermagem na APS.

Precisamos conhecer e agregar experiências locais de práticas de defesa ambiental ecocêntricas como integrante do que-fazer da enfermagem: onde estão e quem são os profissionais que, por exemplo ensinam comunidades a preservar nascentes de água, reconhecendo que a saúde hídrica é vital para prevenir doenças? Onde estão e quem são os profissionais que apoiam movimentos e lutam por leis que reconheçam rios ou florestas como sujeitos de direitos, entendendo que sua “saúde” é condição para a nossa?

Apesar desse potencial, a enfermagem enfrenta obstáculos estruturais para realizar essa visão como: sobrecarga de trabalho, com profissionais submersos em demandas assistenciais e com pouco espaço para atuação política ambiental; formação tecnicista, por meio de grades curriculares que ainda priorizam procedimentos clínicos sobre temas como justiça climática ou ecologia política. Na base das questões que impedem a enfermagem de ampliar sua prática na direção ecocêntrica, está a mercantilização do cuidado, que reduz a saúde a um serviço individual, apagando sua dimensão ecológica.

A *hybris* do Antropoceno nos ensinou que não há saúde humana em um planeta doente. Cabe à enfermagem, como prática social, contribuir para “costurar” essa ruptura, lembrando que ação de cuidado, com cada campanha de vacinação, cada gesto de acolhimento, é também um ato de resistência contra um sistema que envenena rios, solos e corpos^.^ Se a *hybris* é a doença, a enfermagem pode ser, em comunhão e diálogo coletivo com outros profissionais e outros seres, cuidadora de mundos.

Enquanto “curar” pode implicar uma relação de poder (humano sobre a natureza), “cuidar” pressupõe humildade e responsabilidade compartilhada. A enfermagem, como cuidadora de mundos, não busca “salvar” o planeta, mas tecer redes de coexistência que sustentem a vida em todas as suas formas.

Essa abordagem ecoa o pensamento de Donna Haraway, que defende “ficar com o problema” (*staying with the trouble*), ou seja, reconhecer as questões e se envolver em práticas cotidianas de cuidado, mesmo sem garantias de solução, diante das graves ameaças ambientais. Ficar com o problema significa estabelecer elos com outras pessoas e áreas disciplinares, e outros seres, estabelecendo “novos parentescos”. A autora destaca, ainda, a importância da criatividade sustentadora das pessoas - ativistas, artistas, cientistas, comunidades - que se importam e agem^(11)^.

No dizer de Ailton Krenak, líder indígena, “não somos donos da Terra; somos sua extensão”^(7)^. A enfermagem, ao abraçar essa máxima, pode redefinir seu legado: de profissão que cuida de humanos a movimento que defende a vida em todas as suas formas.

## CONSIDERAÇÕES FINAIS

Este texto não busca respostas, mas provoca reflexões a partir de algumas perguntas: como equilibrar a urgência da saúde planetária com a necessidade de desconstruir o antropocentrismo? Se as plantas pensam, quem somos nós para decidir seu destino? A ciência de Mancuso é um espelho que reflete nossa *hybris*, e talvez, nesse reflexo, encontremos a humildade necessária para reexistir.

Adotar a metáfora das plantas pode reconduzir nossa visão de mundo e de prática profissional como um possível guia para um cuidado cooperativo e não exploratório, e para a justiça ambiental. Como enfermeiros, ao buscarmos abraçar essa perspectiva em nossa prática cotidiana, devemos ter a humildade para um aprendizado necessário e contínuo, ampliando nossa escuta. Isso implica assumir nosso papel permanente de educadores populares em uma perspectiva dialógica permanente, na busca de maior qualificação sobre os temas da saúde planetária, no compartilhamento de nossas iniciativas locais, especialmente aquelas que se desenvolvem em diálogo nos diferentes territórios em que estamos inseridos. A partir da liderança que já exercemos como mediadores entre profissões e organizações de saúde, educadores, e formadores, é preciso que nos alinhemos com as pautas que enfrentam as diversas formas de injustiça social - a ambiental incluída.

Concordamos com os autores trazidos nesta reflexão, que afirmam que não adianta declarar guerra ao aquecimento global se continuarmos a guerrear contra a própria Terra. A verdadeira cura não virá de planos de gerenciamento, mas de um despertar ético que nos lembre: somos hóspedes, não donos, deste planeta. E talvez a lição mais urgente seja aprender a ouvir, em vez de comandar, as vozes silenciadas da Terra - incluindo aquelas que nunca estiveram interessadas em nos salvar, mas que certamente têm e terão papel central no futuro da nossa espécie - as plantas.

## Data Availability

Não se aplica.
